# *BAX -248 G>A* and *BCL2 -938 C>A* Variant Lowers the Survival in Patients with Nasopharyngeal Carcinoma and Could be Associated with Tissue-Specific Malignancies: A Multi-Method Approach

**DOI:** 10.31557/APJCP.2021.22.4.1171

**Published:** 2021-04

**Authors:** Koustav Chatterjee, Saikat De, Sankar Deb Roy, Sushil Kumar Sahu, Arindom Chakraborty, Sandeep Ghatak, Nilanjana Das, Sudipa Mal, Nabanita Roy Chattopadhyay, Piyanki Das, R. Rajendra Reddy, Syamantak Mukherjee, Ashok Kumar Das, Zoreng Puii, Eric Zomawia, Yengkhom Indibor Singh, Sam Tsering, Komri Riba, Shanmugam Rajasubramaniam, Amol Ratnakar Suryawanshi, Tathagata Choudhuri

**Affiliations:** 1 *Department of Biotechnology, Visva-Bharati, Santiniketan, Birbhum, West Bengal, India. *; 2 *Department of Radiation Oncology, Eden Medical Centre, Dimapur, Nagaland, India. *; 3 *Department of Pharmacology and Molecular Sciences, School of Medicine, Johns Hopkins University, Baltimore, Maryland, United States. *; 4 *Department of Statistics, Visva-Bharati, Santiniketan, West Bengal, India. *; 5 *Division of Animal and Fishery Science, ICAR Research Complex for North East Hill Region,Umiam, Meghalaya, India. *; 6 *Clinical Proteomics, Institute of Life Sciences, Bhubaneswar, India. *; 7 *Department of ENT, Dr B. Borooah Cancer Institute, Guwahati, Assam, India. *; 8 *State Referral Hospital, Falkawn, Mizoram, India. *; 9 *Regional Institute of Medical Sciences, Department of Radiotherapy, Imphal, Manipur, India. *; 10 *Tertiary cancer center,TomoRiba Institute of Health And Medical Sciences, Arunachal Pradesh, India. *; 11 *Division of Genetic Disorders ICMR-National Institute of Research in Tribal Health, NIRTH Complex, Jabalpur, Madhya Pradesh, India. *

**Keywords:** BAX- BCL2, polymorphism- SNP, nasopharyngeal carcinoma, NPC, computational study, meta-analysis

## Abstract

**Background:**

The association of *BAX -248 G>A* and *BCL2 -938 C>A* with different cancers created conflicts. We studied the correlation and the effect of these polymorphisms in patients with Nasopharyngeal Carcinoma (NPC).

**Methods:**

PCR-RFLP and Sanger sequencing were used to detect polymorphisms. Statistical analysis including forest plot and Kaplan-Meier Log-rank test was conducted to investigate the association and effect of these SNPs on the NPC patients’ survival. The computational study was performed to investigate the possible regulatory role between these polymorphisms and the poor survival of NPC patients. Meta-analysis was executed to check the tissue-specific association of these polymorphisms in the context of global cancer prognosis.

**Results:**

We observed an increased and significant association of *BAX -248 G>A* [GA:OR=5.29, 95%CI=1.67,16.67, P=0.004; GA+AA:OR=5.71, 95%CI=1.82,17.90, P =0.002; A:OR=5.33, 95%CI=1.76,16.13, P=0.003], and *BCL2 -938 C>A* [CA:OR=2.26, 95%CI=1.03,4.96, P=0.04; AA:OR=3.56, 95%CI=0.97,13.05, P=0.05; CA+AA:OR=3.10, 95%CI=1.51,6.35, P=0.002; A:OR=2.90, 95% CI=1.59,5.29, P=0.0005] with the risk of NPC. Also, these SNPs were strongly correlated with poor survival in NPC patients (lower estimated survival mean, lower estimated proportion surviving at 5 years with p<0.05). The computational study showed that these SNPs altered the binding affinity of transcription factors *HIF1*, *SP1, PAX3, PAX9* and *CREB* towards promoter (Lower p indicates strong affinity). The meta-analysis revealed the tissue-specific association of these polymorphisms. *BAX -248 G>A* showed a significant correlation with carcinomas [A vs G:OR=1.60, 95%CI=1.09,2.34, P=0.01; AA vs GG:OR=2.61, 95%CI=1.68,4.06, P<0.001; AA+GA vs GG:OR=1.53,95%CI=1.04,2.25, P=0.02); AA vs GG+GA:OR=2.53, 95%CI=1.65,3.87, P<0.001], and *BCL2 -938 C>A* with other malignancies [A vs C:OR=1.45, 95%CI=1.26,1.66, P<0.001; AA vs CC:OR=2.07, 95%CI: 1.15,3.72, P=0.01; AA+CA vs CC:OR=1.42, 95%CI=1.18,1.72, P<0.001; AA vs CC+CA:OR=1.89, 95%CI=1.02,3.50, P=0.04].

**Conclusions:**

BAX -248 G>A and BCL2 -938 C>A was associated with poor survival in NPC patients. It may increase cancer susceptibility through transcriptional regulation. Moreover, these SNPs’ effects could be tissue-specific.

## Introduction

Genetic polymorphisms make up the basics of phenotypic diversity. Increasing evidence showed substantial sequence abnormalities such as single nucleotide polymorphism (*SNP*) and structural alteration in the individual organism (Manke et al., 2010). SNP is the most studied form of sequence variation, which sometimes may enhance disease susceptibility including cancers (Deng et al., 2017). It is detected in different parts of a gene covering from the untranslated regions to the functional regions. Thus, the effect of SNPs in susceptibility towards diseases may vary depending on the location of such alterations. SNP in the promoter region may alter gene expression either by altering transcription factor (TF) binding, DNA methylation, or histone modifications. However, the functional consequences of SNPs are more challenging to predict and validate, because the regulatory code is much more complex than the genetic code (Manke et al., 2010; Deng et al., 2017). 

So far, promoter polymorphism of *BAX -248 G>A* and *BCL2 -938 C>A* are two of the most studied polymorphisms reported being associated with the increased risk of cancers (Sahu et al., 2013; Yao et al., 2017). *BAX* and *BCL2* are the members of the *BCL-2* family that regulates apoptosis differently. *BAX *(*BCL-2* associated X protein, OMIM 600040) is a pro-apoptotic protein, promotes apoptosis via mitochondrial-mediated pathway. *BCL2 *(OMIM 151430), on the other hand, is categorized as an anti-apoptotic protein that inhibits apoptosis by inhibiting BAX. Though, two proteins are located in different chromosomes (BAX on19q13.33 and BCL2 on 18q21.33), they functionally bind with each other to regulate cell proliferation. Evidence suggested that the expression of these proteins is regulated individually by many other cellular components and that the ratio of their relative levels determines the cell fate (Fernandes et al., 2015; Khodapasand et al., 2015). Thus, any aberration in their expression level may lead to an increase in cell proliferation.

Some findings suggested that these SNPs increased the risk of cancer by altering their gene expressions and lowering the survival in patients with malignancies (Saxena et al., 2002; Cingeetham et al., 2015; Fernandes et al., 2015; Javid et al., 2015 a; Bhushann Meka et al., 2016). For example, *BAX* promoter polymorphism altered its expression in chronic lymphocytic leukemia and significantly shortened the patients’ survival in many cancers (Saxena et al., 2002; Fernandes et al., 2015). Similar results were found in the case of BCL2 (Zenz et al., 2009; Moon et al., 2010). However, SNP observed in specific characteristics may not unavoidably the causative change (Manke et al., 2010). Thus, preliminary data based on the computational study is required to produce an assumption about the regulatory mechanism involved. Moreover, further evidence demonstrated the ethnicity-specific association of these SNPs, as the Asian population is exhibited a significant correlation compared to Caucasians (Zhang et al., 2014; Yao et al., 2017; Feng et al., 2018). Besides the correlation with cancer susceptibility, negative associations are also reported in some malignancies. Hence, conflicts are still there about the association of these polymorphisms with cancer susceptibility. Early evidence suggested that the effect of some SNPs could be Tissue-specific. So far, no such association was reported in *BAX -248 G>A* and *BCL2 -938 C>A* polymorphism. Thus for better rationalization, the association and the effect of these polymorphisms on different cancer susceptibility need to be inspected in any ethnicity-specified and tissue-specific cancers. 

Therefore, in our present study, efforts were made to explore the correlation and effect of *BAX -248 G>A* and *BCL2 -938 C>A SNPs* on nasopharyngeal carcinoma (NPC) patients’ survival. Further, the impact of these polymorphisms in different tissue-specific (carcinoma vs other malignancies) cancers was investigated. NPC was chosen for several reasons, unlike other cancers, the occurrence and distribution of NPC depend on the ethnic variation. In Asia, it is uniquely prevalent in southern China, Indonesia, and Northeastern India (NEI) (Kataki et al., 2011; Chua et al., 2016). Moreover, about 20% to 30% of the NPC patients with the same stages, receiving similar treatment, showed local relapse or distal metastasis, indicating the involvement of genetic factors (Chen et al., 2012; Jiang et al., 2015). Moreover, the correlation of these SNPs with NPC prognosis is still unknown. 

## Materials and Methods

*Study selection*

NPC samples (n=100) were collected from different state hospitals of NEI between 2014 and 2018. For each subject, epidemiological and clinicopathological information was verified and considered for the study. Based on the instructions of the World Health Organization (WHO), clinical examinations of all cases were confirmed (Sahu et al., 2016). NPC stages were determined according to the AJCC (American Joint Committee on Cancer) classification system (Amin et al., 2017). For the control group, samples were collected (n=70) from the age and sex-matched healthy volunteers of the same ethnic background. According to the research review committee guideline, informed consent was obtained from all subjects. The characteristics of NPC patients and controls are described in supplementary table S1. The present study was approved by the Institutional ethics committee. Also, ethical approval was obtained from all the participating institutes.

*Detection of genotypes by PCR-RFLP and Sequencing*

Genomic DNA was extracted from each sample using the genomic DNA extraction kit (Invitrogen, CA, US) following the manufacturer’s instructions. Initially, eight NPC samples were removed from the study due to poor DNA content. A total of 92 NPC samples and 68 healthy controls were used to identify BCL2 -938 C>A. After the first analysis, 22 samples containing an insufficient amount of DNA were eliminated and 70 NPC samples and 70 healthy controls were considered further for the detection of BAX -248 G>A. Gene symbols were verified according to the nomenclature guidelines of the Human Genome Variation Society (HGVS) (den Dunnen et al., 2016). Promoter regions of both *BAX* and *BCL2 *were amplified by PCR with specific primers as previously reported (Starczynski et al., 2005; Zhang et al., 2011). For restriction fragment length polymorphism (RFLP), PCR products were purified and half of the volume was digested by TauI (Thermo Fisher, MA, US) and BccI (NEB, MA, US) to identify genotypes of BAX and BCL2 respectively (Starczynski et al., 2005; Zhang et al., 2011). Another half of the purified PCR products were sent to the service provider (Chromous Biotech, Bengaluru, India) for Sanger sequencing to cross-check the previous analysis result. After the sequencing, sequences were aligned with the reference sequence for BAX (rs 4645878) and BCL2 (rs2279115) using the CLUSTALW (https://www.genome.jp/tools-bin/clustalw) to check sequence similarity.

*Study the genetic association and NPC patients’ survival*

The allele and genotype frequencies in NPC cases and healthy controls were calculated manually (Sahu et al., 2016). Linear regression was measured by Fisher’s exact test to investigate the association of *BAX -248 G>A*, and *BCL2 -938 C>A* polymorphism with the risk of NPC (Fisher, 1922). Odds ratio (OR) was calculated for each genetic model to show the association between the exposure and outcome, 95% of Confidence Interval (CI) was analyzed to determine the population mean precisely, and the p-value was measured for the level of significance, where P<0.05 was considered as significant. A fully nonparametric Kaplan-Meier analysis was conducted to test the survival probability due to SNPs. Log-Rank test was used to estimate if there were a statistically significant difference in the cumulative proportions across groups. All the patients’ data was arranged according to the genotype combination. Endpoint or censored information of each patient was obtained from the hospital cancer registry. MedCalc Statistical Software, version 18.1 (MedCalc Software, Ostend, Belgium), and R studio (Version 1.3) were used for the statistical calculation (Mavrogenis et al., 2012). 

*Functional analysis of BAX -248 G>A and BCL2 -938 C>A*

The computational approach was performed to investigate the possible regulatory mechanism involved due to these polymorphisms in terms of transcription factors (TFs) binding to the specific polymorphic region. The difference of TFs’ binding affinity was measured using the online available tool named sTRAP (http://trap.molgen.mpg.de/cgi-bin/trap_two_seq_form.cgi), which predict TFs binding affinity changes to a region (Manke et al., 2010). The sTRAP is an affinity-based method, uses both TRANSFAC and JASPAR databases to search the query. It has several advantages over other hit-based approaches that use position-specific scoring matrices (PSSMs) (Thomas-Chollier et al., 2011). After initial analysis for the TFs, weight scores were measured from the prior chosen weight matrices. Then total affinity of a sequence for a TF was calculated by affinity values (p-value), where a lower p-value indicated a strong affinity (Manke et al., 2010; Thomas-Chollier et al., 2011).

*Study the impact of BAX and BCL2 SNPs on tissue-specific malignancies*

Meta-analysis was conducted to investigate if there is any impact of these SNPs on tissue-specific cancers. Qualified publications were retrieved from publicly available Medline databases (PubMed), EMBASE, Science Direct, and Wiley online library until October 2018. To assess the quality of the meta-analysis, the method of each study was considered independently following the PRISMA statement (Liberati et al., 2009). Initially, 766 reports were identified from various databases and 3 articles from other sources were included to make our search inclusive. Following the removal of duplicates, 312 records were identified. After a full-length screening, studies containing irrelevant data, missing information, etc. were excluded, and eventually, 29 studies were considered. Our current case-control study was also included, to increase the study size (Supplementary Figure S1). Hardy–Weinberg equilibrium (HWE) was calculated to detect a deviation in genotype distribution of the control groups using the web-based tool (https://wpcalc.com/en/equilibrium-hardy-weinberg and https://www.graphpad.com/quickcalcs/PValue1.cfm). P >0.05 was considered as no deviation from the Hardy–Weinberg equilibrium. Forest plot analysis was performed to evaluate the association in 5 different models, i.e. allelic (A vs G/A vs C); homozygous (AA vs GG/AA vs CC); heterozygous (GA vs GG/CA vs AA); dominant (AA+GA vs GG/ AA+CA vs CC) and recessive model (AA vs GG+GA/ AA vs CC+CA). 

## Results

*Detection of BAX -248 G>A and BCL2 -938 C>A and association with NPC patients’ survival*

**Figure 1:**
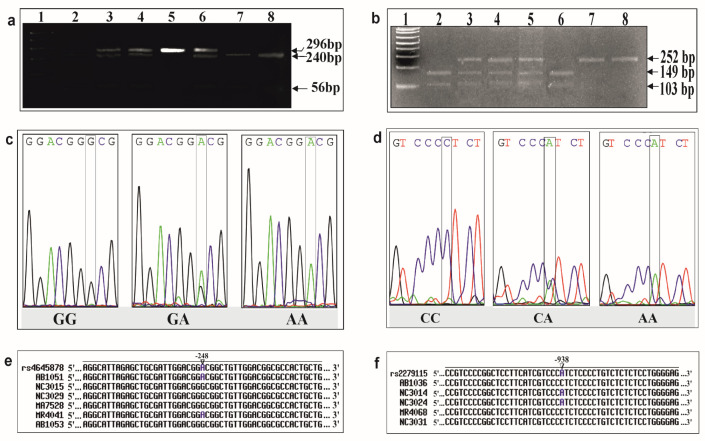
Genotype Detection of BAX and BCL2 in NPC. Gel electrophoresis image from PCR-RFLP analysis (a) showed different band patterns of BAX genotypes: three bands (296 bp undigested and 240 bp + 56 bp digested) indicated GA (lane 3, 4, and 6), two bands (240 bp + 56 bp digested), indicated GG genotype (lane 2, 7, and 8); and a single band of 296 bp indicated AA. Gel electrophoresis image (b) showed different band patterns of BCL2 genotypes: three types of band patterns. lane3, 4, and 5 showed three bands (252 bp undigested and 149 bp + 103 bp digested), indicated CA; lane 2 and 6 showed two bands (149 bp + 103 bp digested) indicated AA, and lane7 and 8 showed a single band of 252 bp, indicated CC. The sequencing chromatogram revealed the consistency of the previous PCR-RFLP study of BAX and BCL2 (c & d). Sequence similarity of the BAX (e) and BCL2 (f) sequences with their reference sequences (BAX: rs 4645878; BCL2: rs2279115) using multiple sequence alignment

The results of the PCR-RFLP and sequencing for the detection of BAX and BCL2 genotypes is shown in figure 1. Among the NPC cases, 74.28%, 24.28% and 1.42% were GG, GA and AA positive respectively. Likewise, in BCL2 SNP, 55.43%, 30.43% and 14.13% were CC, CA and AA positive respectively (Table 1). The association study indicated that genotype GA, GA+AA and allele A were significantly increased the risk of NPC [GA: (OR=5.29, 95% CI= 1.67, 16.67, P=0.004); GA+AA: (OR = 5.71, 95% CI = 1.82, 17.90, P = 0.002); A: (OR=5.33, 95% CI=1.76, 16.13, P=0.003)]. Similarly, a significant correlation between *BCL2 -938 C>A* polymorphism and NPC was found in the genotype CA, AA, CA+AA and the allele A [CA: (OR= 2.26, 95% CI= 1.03, 4.96, P= 0.04); AA: (OR= 3.56, 95% CI= 0.97, 13.05, P= 0.05); CA+AA: (OR=3.10, 95% CI= 1.51, 6.35, P=0.002); A: (OR=2.90, 95% CI= 1.59, 5.29, P=0.0005)]. BAX GA+ BCL2 CA dual positive genotype was also observed in seven (10%) NPC cases and one (1.42%) healthy control. Genetic association study showed that dual positive heterozygote was not significantly associated with the risk of NPC (Table 1). 

**Figure 2:**
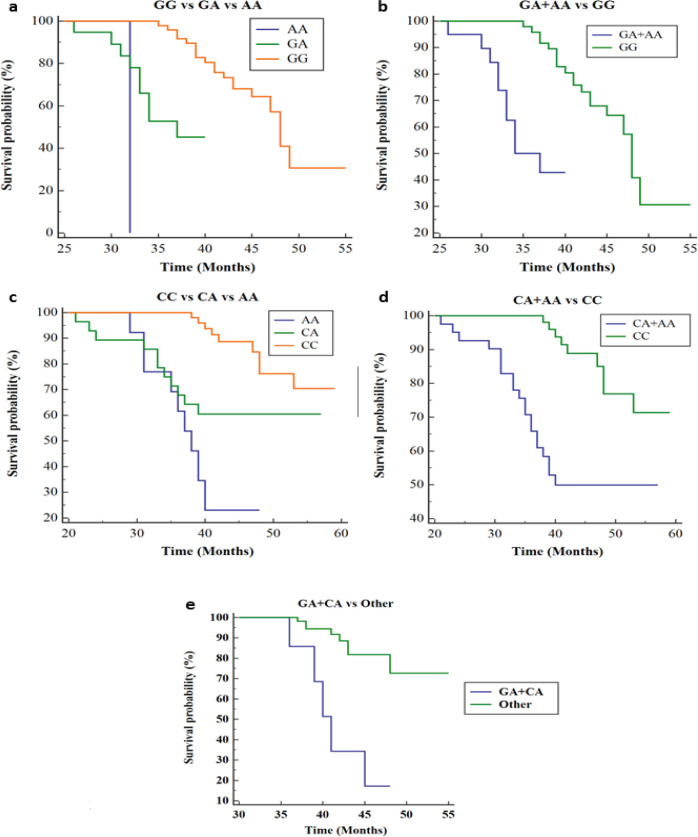
Kaplan-Meier Survival Analysis. Plot a and b indicated survival probabilities of GG vs GA vs AA and GA+AA vs GG respectively. Plot c and d indicated survival probabilities of CC vs CA vs AA, and CA+AA vs CC respectively. Plot e described the survival probabilities in patients containing both GA+CA heterozygote vs other

The result of the Kaplan-Meier survival analysis and log-rank test indicated that the dominant model of *BAX* (GA+AA) and* BCL2* (CA, AA, and CA+AA) polymorphisms are the independent predictors for the lower survival in patients with NPC. Likewise, the dual positive heterozygote of BAX and BCL2 have demonstrated decreased survival. Also, the same genetic models for *BAX, BCL2*, and dual positive heterozygote showed the lowest Proportion of Surviving at 5 years. Due to the detection of only one AA genotype in BAX G>A, the true effect of GA vs AA vs GG on survival was not observed (Figure 2 and Supplementary Table S2).

*Computational analysis for the possible functional effect of BAX -248 G>A and BCL2 -938 C>A*

**Figure 3:**
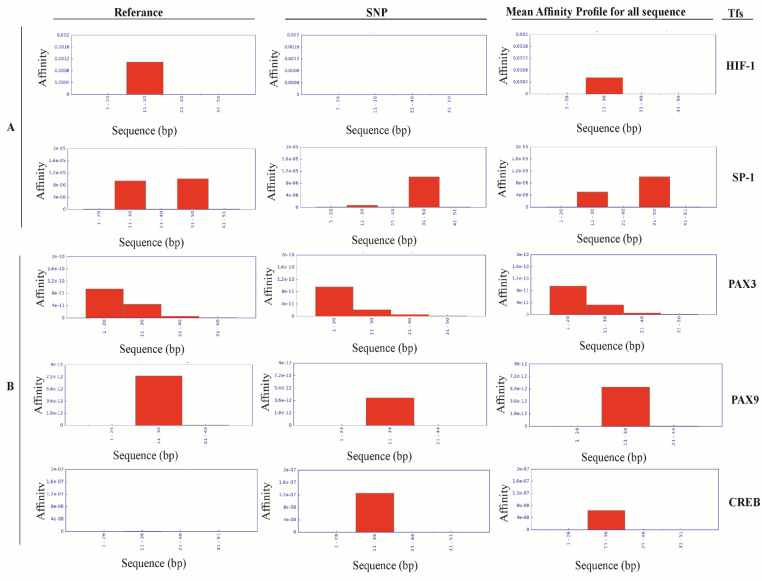
Affinity Plot for Different Transcription Factors (TFs) Binding. Panel A represented the TFs binding affinity changes at the polymorphic region of *BAX* (*BAX* (-248) G>A) vs normal. Panel B indicated the TFs binding affinity changes at the polymorphic region of* BCL2* (*BCL2* (-938) C>A) vs normal. The X- axis of each plot denoted the size (in bp) of the sliding window used for the affinity calculations and affinity graphs, which is smaller than the DNA sequence length, whereas, the Y-axis signified the affinity values in logarithmic exponential function (e^x^).

We only considered TFs, those have previous reports on the interaction with BAX and BCL2 mediated apoptosis regulation. The results illustrated that binding affinity of Hypoxia Inducing factor 1 (HIF1) and SP1 to the BAX promoter is altered after the *BAX -248 G>A *polymorphism [HIF1: (Pref =0.066, PSNP =0.547); SP1: (Pref =0.687; PSNP =0.726)]. On the other hand, *BCL2 -938 C>A *polymorphism changed the binding affinity of PAX3, PAX9 and CREB to the promoter [PAX3: (Pref =0.089; PSNP = 0.006); PAX9: (Pref =0.063; PSNP = 0.338); CREB (Pref= 0.465; PSNP= 0.082) (Figure 3 and supplementary Table S3). 

*The impact of BAX -248 G>A (rs 4645878) on tissue-specific malignancies*

**Figure 4:**
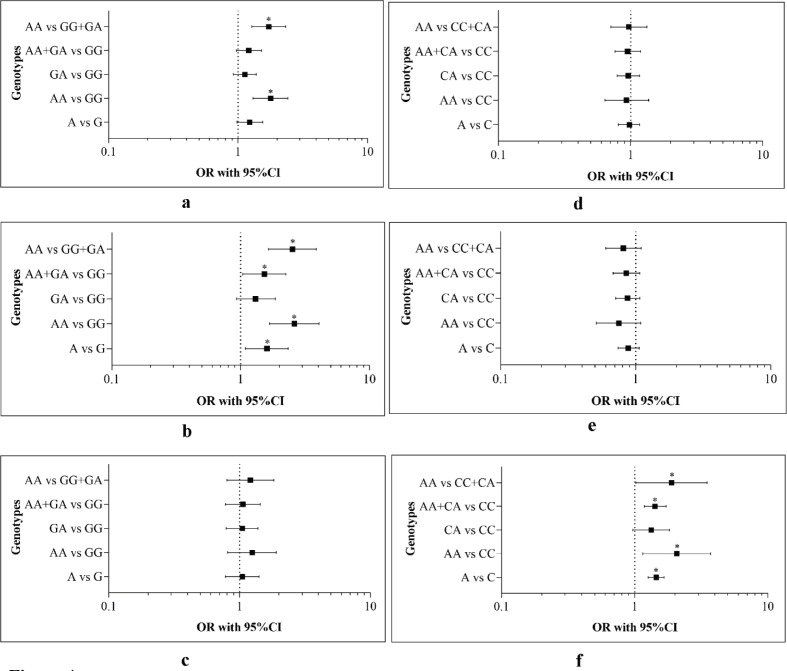
Forest Plot in Meta-Analysis. Plot a and d showed the association of *BAX* (-248) G>A and *BCL2* (-938) C>A with overall cancer susceptibility. Plot b and e showed the association of *BAX* (-248) G>A and *BCL2* (-938) C>A with carcinomas. Plot c and f signified the correlation of *BAX* (-248) G>A and *BCL2* (-938) C>A with other malignancies. The odds ratios (OR) are represented by the square and the 95% CIs are denoted by horizontal lines. Significant p-values (<0.05) are indicated by an asterisk

A total of 15 case-control studies (including 3460 cases and 3404 controls) of BAX -248 G>A SNP and cancer risk were included in the meta-analysis (Table 2). The result of the overall study and subgroup analyses is shown in figure 4 and supplementary table S4. Overall study showed a correlation with the susceptibility of cancer in AA vs GG (OR= 1.79, 95% CI= 1.31, 2.43, P<0.001) and AA vs GG+GA (OR= 1.73, 95% CI= 1.28, 2.33, P<0.001) genetic models. No association was observed from the rest of the genetic models (Figure 4a). Result of the stratified analysis indicated that *BAX* (rs 4645878) polymorphism was significantly associated with carcinomas in four genetic models [A vs G: (OR= 1.60, 95% CI= 1.09, 2.34, P= 0.01); AA vs GG: (OR= 2.61, 95% CI= 1.68, 4.06, P<0.001); AA+GA vs GG: (OR= 1.53, 95% CI= 1.04, 2.25, P= 0.02); AA vs GG+GA: (OR= 2.53, 95% CI= 1.65, 3.87, P<0.001)] (Figure 4b). The rest of the group showed no significant association (Figure 4c). HWE analysis demonstrated that the control group did not deviate from the Hardy–Weinberg equilibrium except for two studies (Supplementary Table S5).

*The impact of BCL2 -938 C>A (Rs2279115) on tissue-specific malignancies*

Figure 4 and Supplementary Table S6 displayed the result of the tissue-specific impact of* BCL2 -938 C>A* polymorphism on global cancer susceptibility in both overall and stratified analysis. 16 studies (including 5471 cases and 5,972 controls) were identified for the study (Table 3). No statistically significant correlation was found between BCL2 (rs2279115) and overall cancer risk (Figure 4d). The result of the subgroup analysis exhibited that the this polymorphism was significantly correlated with the susceptibility of other malignancies in four genetic models [A vs C: (OR= 1.45, 95% CI= 1.26, 1.66, P<0.001); AA vs CC: (OR= 2.07, 95% CI= 1.15, 3.72, P= 0.01); AA+CA vs CC: (OR= 1.42, 95% CI= 1.18, 1.72, P<0.001); AA vs CC+CA: (OR= 1.89, 95% CI= 1.02, 3.50, P= 0.04)] (Figure 4e). No significant association was observed in carcinomas (Figure 4f). Results of the HWE analysis indicated that no such deviations occurred in most of the studies except a few (Supplementary Table S7).

*Study of heterogeneity, sensitivity analysis, publication bias, minor allele frequency, and post hoc power*

The Minor allele frequency (MAF) was calculated for each of the studies to rationalize the difference between common and rare variants (supplementary Table S5 and S7). Heterogeneity with Q value, P-value, and I square statistics were assessed for the individual model. The random (Der Simonian and Laird method) or ﬁxed (Mantel–Haenszel’s method) model was used to calculate the combined OR and 95% CI based on heterogeneity or homogeneity among studies. The random-effect model was chosen if the Q statistic was significant (p <0.05). In the meta-analysis, Substantial heterogeneity was observed (I^2^ >50%) in the overall studies for BAX (rs 4645878) and BCL2 (rs2279115). Genetic models A vs G, GA vs GG, and AA+GA vs GG for BAX (rs 4645878) showed a significant source of heterogeneity (p< 0.05). Likewise, a significant level of heterogeneity was observed in any of the five genetic models of the overall study in BCL2 (rs2279115). Identification of the source of heterogeneity in the subgroup analysis was difficult due to the effect of a small number of studies (Supplementary Table S4 and S6). To assess the stability of the results, the sensitivity analysis was performed individually for *BAX* and *BCL2* polymorphisms in the overall meta-analysis by ignoring one study at a time and then computing the pooled ORs again (Supplementary Figure S2 and S3). The result pointed out that the meta-analysis was not influenced by any single study in any of the five genetic models of the *BAX* polymorphism in the overall study [A vs G: (OR=1.23, 95% CI=0.98, 1.55, p=0.06); AA vs GG: (OR=1.79, 95% CI=1.31, 2.43, p=0.00); GA vs GG: (OR=1.13, 95% CI=0.92, 1.38, p=0.22); AA+GA vs GG: (OR=1.21, 95% CI=0.97, 1.52, p=0.09); AA vs GG+GA: (OR=1.73, 95% CI=1.28, 2.33, p=0.00)]. Similar finding was observed in the case of *BCL2* polymorphism [A vs C: (OR=0.98, 95% CI=0.81, 1.17, p=0.83); AA vs CC: (OR=0.93, 95% CI=0.64, 1.37, p=0.74); CA vs CC: (OR=0.96, 95% CI=0.79, 1.17, p=0.74); AA+CA vs CC: (OR=0.95, 95% CI=0.76, 1.19, p=0.70); AA vs CC+CA: (OR=0.97, 95% CI=0.71, 1.33, p=0.88)], thus our analysis was robust and stable. Funnel plot and Egger regression analyses were performed to designate the publication bias for each of the five genetic models of the overall study. Eggers test revealed that all p values were >0.05 and funnel plots were relatively symmetrical in any of the genetic models of the meta-analysis which indicated the absence of publication bias in the current study (Supplementary Figure S4). Statistical post hoc power was calculated assuming a small effect size (w=0.15) to measure the probability of the presence of any small effect in the study. Results depicted that studies containing low study size shown weak power (<0.5) to detect mild effects of the polymorphisms on disease susceptibility (Table 2 and 3). 

**Table 1:** Genotype and Allele Distribution of *BAX* (-248) G>A and *BCL2 *(-938) C>A and Association with NPC Prognosis

SNPs	Genotype	Healthy Control (%)	NPC Case (%)	OR (95% CI)	P-value
*BAX* (-248) G>A (rs 4645878) Control/Case =70/70	GG	66 (94.28)	52 (74.28)	Ref.	
	GA	4 (5.71)	17 (24.28)	5.29 (1.67, 16.67)	0.004
	AA	0 (0.00)	1 (1.42)	3.04 (0.12, 75.99)	0.49
	GA+AA	4 (5.71)	18 (25.71)	5.71 (1.82 to 17.90)	0.002
	GG	66 (94.28)	52 (74.28)	Ref.	
	GG-GA	70 (100.00)	69 (98.57)	Ref.	
	AA	0 (0.00)	1 (1.42)	3.04 (0.12, 75.99)	0.49
	G	136 (97.14)	121 (86.42)	Ref	
	A	4 (2.85)	19 (13.57)	5.33 (1.76 , 16.13)	0.003
*BCL2*(-938) C>A (rs2279115) Control/Case =68/92	CC	54 (79.41)	51 (55.43)	Ref.	
	CA	11(16.17)	28 (30.43)	2.26 (1.03 , 4.96)	0.04
	AA	3 (4.41)	13 (14.13)	3.56 (0.97 , 13.05)	0.05
	CA+AA	14 (20.58)	41 (44.56)	3.10 (1.51 , 6.35)	0.002
	CC	54 (79.41)	51 (55.43)	Ref.	
	CC-CA	65 (95.58)	79 (85.86)	Ref.	
	AA	3 (4.41)	13 (14.13)	3.56 (0.97 , 13.05)	0.05
	C	119 (87.5)	130 (70.65)	Ref.	
	A	17 (12.5)	54 (29.34)	2.90 (1.59 , 5.29)	0.0005
BAX (-248) G>A & *BCL2*(-938) C>A dual positive	GA+CA	1 (1.42)	7 (10.00)	7.66 (0.91, 64.06)	0.06

SNPs, Single Nucleotide Polymorphisms; OR, Odds Ratio; 95% CI, Confidence Interval; rs 4645878, reference no of *BAX* (-248) G>A; rs2279115, reference no of *BCL2* (-938) C>A in the NCBI database.

**Table 2:** Reports Included in the Meta-Analysis of* BAX* (-248) G>A Polymorphism and Cancer Susceptibility

Study	Country	Ethnicity	Cancer type	Study design	Genotype method	Sample size		Power (%)†
						Control	Case	
Chen K et al, 2007 (Chen et al., 2007)	USA	Caucasian	SCC	HB	PIRA-PCR	934	814	99.5
Cingeetham A et al, 2015 (Cingeetham et al., 2015)	India	Asia	AML	HB	PCR-RFLP	305	218	74.5
Dholariya et al 2016 (Dholariya et al., 2016)	India	Asia	EOC	PB	PIRA–PCR	70	70	24.1
Edathara P.M et al, 2016 (Edathara et al., 2016)	India	Asia	CML	HB	PCR-RFLP	509	477	92.3
Javid J et al, 2015 (Javid et al., 2015 a)	India	Asia	NSCLC	HB	PIRA-PCR	160	160	47.5
Mirmajidi H. et al, 2015 (Mirmajidi et al., 2016)	Iran	Caucasian	GC	PB	PCR-RFLP	89	100	29.3
Moazami-Goudarzi et al., 2016 (Moazami-Goudarzi et al., 2016)	Iran	Caucasian	ALL	PB	PCR	62	62	21.8
Nuckel H et. Al, 2006 (Nückel et al., 2006)	Germany	Caucasian	CLL	HB	PCR	95	112	30.9
Oliveira C et al, 2014 (Oliveira et al., 2014)	Brazil	Caucasian	CM	HB	PCR-RFLP	215	200	59.5
Present study, 2018	India	Asia	NPC	HB	PCR	70	70	24.1
Saxena A et al, 2002 (Saxena et al., 2002)	Canada	Caucasian	CLL	HB	PCR	25	34	11.6
Skogsberg A et al, 2006 (Skogsberg et al., 2006)	Sweden	Caucasian	CLL	HB	PCR	207	463	57.8
Starczynski J et al, 2005 (Starczynski et al., 2005)	UK	Caucasian	CLL	HB	PCR	135	203	41.4
Wang WL et al, 2014 (Wang et al., 2014)	China	Asian	NHL	PB	PCR-RFLP	446	424	88.6
Yildiz Y et al, 2013 (Yildiz et al., 2013)	China	Asian	BC	HB	PCR	82	53	27.4

HB, hospital-based; PB, population-based; PCR, polymerase chain reaction; PIRA, Primer introduce restriction analysis; RFLP, restriction fragment length polymorphism; SCC, Squamous Cell Carcinoma; AML, Acute Myeloid Leukemia; EOC, Epithelial Ovarian Cancer; CML, Chronic Myeloid Leukemia; NSCLC, Non-Small-Cell Lung Cancer; GC, Gastric Cancer; ALL, Acute Lymphocytic Leukemia; CLL, Chronic Lymphocytic Leukemia; CM, Cutaneous Melanoma; NPC, Nasopharyngeal Carcinoma; NHL, Non- Hodgkin Lymphoma; BC, Breast Cancer; †, Statistical post hoc power was presented in %

**Table 3:** Reports included in the Meta-Analysis of* BCL2* (-938) C>A Polymorphism and Cancer Susceptibility

Study	country	Ethnicity	Cancer type	Study design	Genotype method	Sample Size		Power (%)†
						Control	Case	
Bachmann HS et al, 2007 (Bachmann et al., 2007)	Germany	Caucasian	LNNIBC	PB	Slowdown PCR	120	274	37.6
Christian DF et al, 2010 (Fingas et al., 2010)	Germany	Caucasian	CCC	HB	PCR	40	40	15.18
Cingeetham A et al, 2015 (Cingeetham et al., 2015)	India	Asia	AML	HB	PCR-RFLP	305	221	74.5
Hirata H et al, 2008 (Hirata et al., 2009)	USA	Caucasian	RC	HB	PCR-RFLP	209	216	58.3
Javid J et al, 2015 (Javid et al., 2015 b)	India	Asia	NSCLC	HB	PIRA-PCR	155	155	46.3
Lehnerdt GF et al, 2009 (Lehnerdt et al., 2009)	Germany	Caucasian	OSCC	HB	Slowdown PCR	150	133	45.1
Li W et al, 2014 (Li et al., 2014)	China	Asia	Glioma	PB	PCR-RFLP	252	248	66.3
Meka PB et al, 2015 (bhushann Meka et al., 2016)	India	Asia	BC	HB	PCR-RFLP	204	110	57.2
Moghaddam E et al, 2017 (Moghaddam et al., 2017)	Iran	Caucasian	BC	HB	PCR	130	120	40.1
Mou X et al, 2015 (Mou et al., 2015)	China	Asia	GC	HB	PCR	129	200	39.9
Pan W et al, 2015 (Pan et al., 2015)	China	Asia	ESCC	HB	PCR-RFLP	1600	1587	99.9
Present study, 2018	India	Asia	NPC	HB	PCR-RFLP	68	92	23.5
Wang WL et al, 2014 (Wang et al., 2014)	China	Asia	NHL	PB	PCR-RFLP	446	424	88.6
Xu P et al, 2013 (Xu et al., 2013)	China	Asia	LC	HB	TaqMan assay	1017	1017	99.8
Yang X et al, 2016 (Yang et al., 2016)	china	Asia	SCLC	HB	Mass Array	1040	520	99.7
Zhang N et al, 2011 (Zhang et al., 2011)	China	Asia	BC	HB	PCR-RFLP	107	114	34.2

HB, hospital-based; PB, population-based; PCR, polymerase chain reaction; PIRA, Primer introduce restriction analysis; RFLP, restriction fragment length polymorphism; LNNIBC, Lymph Node-Negative Invasive Breast Cancer; CCC, Cholangiocellular Carcinoma; AML, Acute Myeloid Leukemia; RC, Renal Cancer; OSCC, Oropharyngeal Squamous Cell Carcinoma; ESCC, Esophageal Squamous Cell Carcinoma; LC, Lung Cancer; SCLC, Small Cell Lung Cancer; NSCLC, Non-Small-Cell Lung Cancer; GC, Gastric Cancer; NPC, Nasopharyngeal Carcinoma; NHL, Non- Hodgkin Lymphoma; BC, Breast Cancer; †, Statistical post hoc power was presented in %.

**Figure 5:**
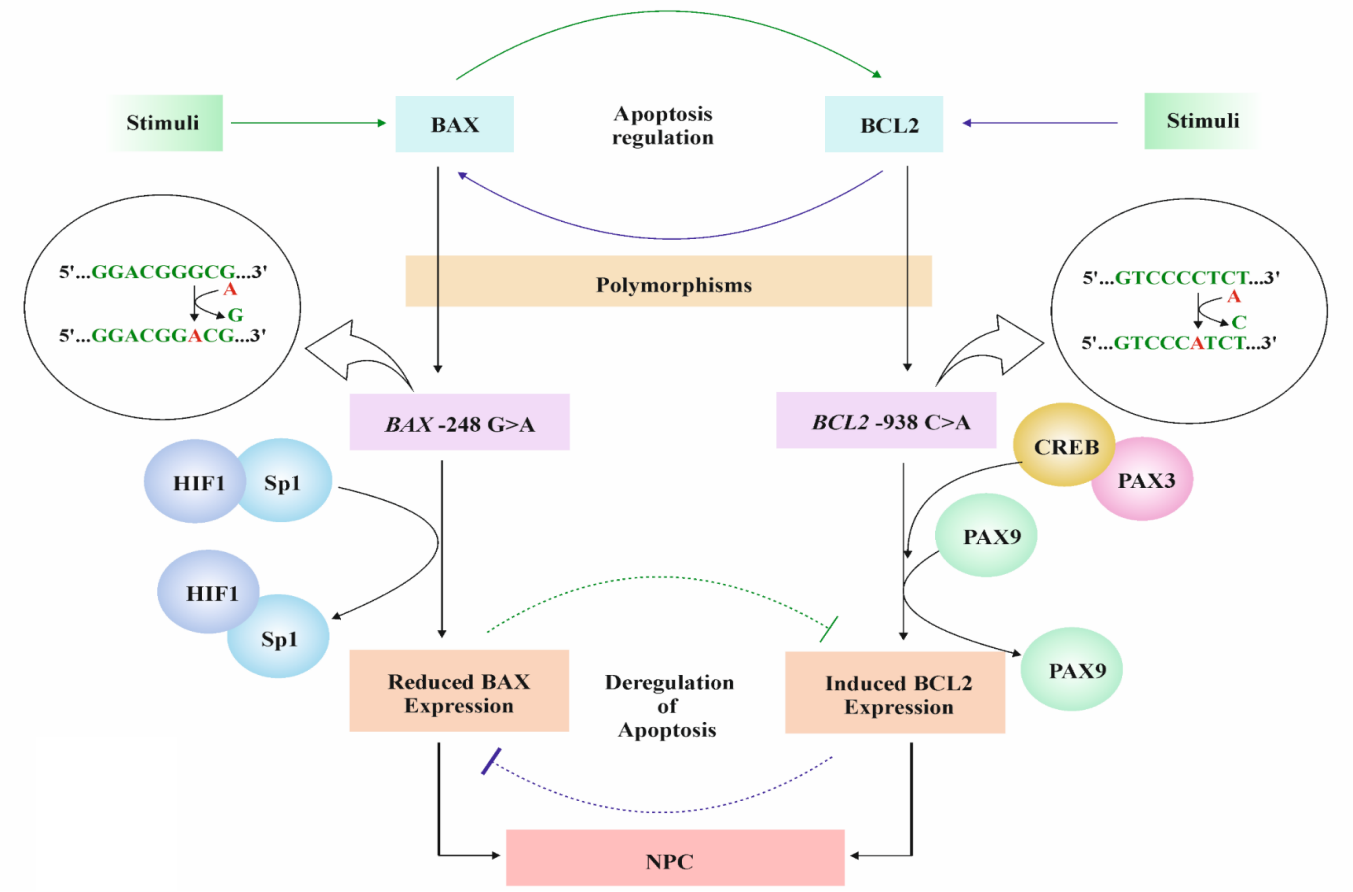
Hypothetical Model of the Effect of *BAX* and *BCL2* Promoter Polymorphism and NPC Susceptibility

## Discussion

In the present study, the genetic association showed that homozygote AA and heterozygote GA in BAX -248 G>A increased the risk of NPC. A similar result was found in the case of BCL2 -938 C>A, where heterozygote (CA) and rare homozygote (AA) was shown to be associated with higher NPC prognosis. Our study was consistent with previous ethnicity-based studies. Further, the presence of both SNPs in the same patient with a higher proportion was noted. Survival curve analysis by Kaplan-Meier and log-rank test suggested that combined dominant genotypes of BAX -248 G>A and BCL2 -938 C>A decreased NPC patients’ survival. The presence of dual SNPs also exhibited the equivalent result.

Computational analysis showed that *BAX* promoter polymorphism might alter the binding affinity of Hypoxia Inducing factor 1 (HIF1) and SP1. HIF1 has a complex role in hypoxia-induced apoptosis by either the activation of p53-mediated cell death or by inducing BNIP3 mediated apoptosis (Greijer et al., 2004). HIF1 down-regulates the BAK and BAX in the tumor through p53 dependent and independent manner (Erler et al., 2004). Unlike HIF1, the interaction of SP1 with the BAX promoter element was investigated, which discovered one additional GC rich SP1 binding site of about six base-pair. Further, mutation analysis in the GC box confirmed that this region is essential for p53 dependent activation (Thornborrow et al., 2001). 

On the other side, BCL2 -938 C>A might change the binding affinity of PAX 9, PAX3, and CREB to the promoter. PAX gene family, including PAX3 and PAX9, plays the opposite role in BCL2 mediated apoptosis through the increase or decrease of its expression (Lee et al., 2008; Hirai et al., 2010; Tan et al., 2017; Arasu et al., 2018). Another TF, called cAMP response element-binding protein (CREB) binds at the upstream promoter of BCL2 and up-regulates its expression (Wilson et al., 1996). Thus, polymorphisms in *BAX -248 G>A* and *BCL2 -938 C>A* may lead to the changes in transcription factor binding towards the promoter, resulting in the altercation of gene expressions, which could be correlated with poor prognosis of the NPC patients’ survival. From the overall study, we established a hypothetical model, which described the effect of these polymorphisms on the onset of NPC prognosis (Figure 5). 

In Meta-analysis, no significant association in the overall study was found. Subgroup analysis of rs 4645878 explored that four genetic models under carcinomas showed a significantly increased risk of cancer than other subgroups. An opposite result was observed in the case of rs2279115, where the subgroup other malignancies displayed a significant association with cancer risk than other subgroups. Previously reported meta-analysis suggested that BAX -248 G>A and BCL2 -938 C>A are not directly associated with the overall cancer susceptibility but the correlation could be ethnicity-specific, which probably contributes to increasing adverse prognosis of cancer (Sahu et al., 2013; Zhang et al., 2014; Yao et al., 2017; Feng et al., 2018). Early reports indicated the tissue-specific effect of several SNPs, but the impact of these two polymorphisms in different cancer types was still unknown. Our study was reliable with the tissue-specific association of *BAX -248 G>A* and* BCL2 -938 C>A* polymorphisms in the context of the global cancer prognosis. 

Though our study was stable as compared to the early findings, some limitations still exist. First, relatively low sample size was used, which might affect the statistical analysis. Second, somewhat few studies were included in the meta-analysis i.e. only case-control reports were considered. Therefore, we could not able to calculate the heterogeneity and publication bias of the subgroup. Finally, due to the unavailability of suitable computational tools, a single web-based tool was used for computational analyses. No other techniques were involved for the cross-validation, even though this is our first prediction and a well-referred tool was used for the approach (Thomas-Chollier et al., 2011; Deplancke et al., 2016; Kumar et al., 2017). 

In conclusion, our findings indicated that *BAX -248 G>A* and *BCL2 -938 C>A *polymorphisms were significantly associated with the susceptibility towards NPC and it lowers NPC patients’ survival. The computational study pointed out that these polymorphisms might affect TFs’ binding. Meta-analysis explained the tissue-specific association of these polymorphisms in regards to the global cancer prognosis.

## Author Contribution Statement

Koustav Chatterjee: Conceptualization; Data curation; Formal analysis; Methodology; Resources; Software; Validation; Visualization; Roles/Writing - original draft. Saikat De: Data curation; Formal analysis. Sankar Deb Roy: Resources. Arindom Chakraborty: Software, Formal analysis, Roles/Writing - original draft. Sandeep Ghatak: Formal analysis, Writing - review & editing. Sushil Kumar Sahu: Methodology, Writing - review & editing. Nilanjana Das: Data curation. Sudipa Mal: Formal analysis. Nabanita Roy Chattopadhyay: Writing - review & editing, Piyanki Das: Writing - review & editing. R Rajendra Reddy: Resources, Data curation. Syamantak Mukherjee: Data curation; Formal analysis. Ashok Kumar Das: Resources. Zoreng puii: Resources. Eric Zomawia: Resources. Y Indibor Singh: Resources. Sam Tsering: Resources. Komri Riba: Resources. Shanmugam Rajasubramaniam: Resources. Amol R Suryawanshi: Resources. Tathagata Choudhuri: Funding acquisition; Investigation; Methodology; Project administration; Supervision; Validation; Visualization; Writing - review & editing.
